# Exploring Rapid and Effective Screening Methods for Anti-SARS-CoV-2 Neutralizing Antibodies in COVID-19 Convalescent Patients and Longitudinal Vaccinated Populations

**DOI:** 10.3390/pathogens11020171

**Published:** 2022-01-27

**Authors:** Caiqin Hu, Dan Li, Zhanmou Liu, Li Ren, Junwei Su, Meiling Zhu, Yi Feng, Zheng Wang, Qiang Liu, Biao Zhu, Yiming Shao

**Affiliations:** 1 State Key Laboratory for Diagnosis and Treatment of Infectious Diseases, National Clinical Research Center for Infectious Diseases, National Medical Center for Infectious Diseases, Collaborative Innovation Center for Diagnosis and Treatment of Infectious Diseases, The First Affiliated Hospital, Zhejiang University School of Medicine, Hangzhou 310003, China; hucaiqin@zju.edu.cn (C.H.); zjusujunwei@zju.edu.cn (J.S.); 2 State Key Laboratory for Infectious Disease Prevention and Control, National Center for AIDS/STD Control and Prevention, Chinese Center for Disease Control and Prevention, Beijing 102206, China; li.ren@chinaaids.cn (L.R.); zhumeiling23@chinaaids.cn (M.Z.); fengyi@chinaaids.cn (Y.F.); wangzheng@chinaaids.cn (Z.W.); 3 Guangxi Key Laboratory of AIDS Prevention and Treatment & Guangxi Universities Key Laboratory of Prevention and Control of Highly Prevalent Disease, School of Public Health, Guangxi Medical University, Nanning 530021, China; michael92752@gmail.com; 4 Gobond Testing Technology (Beijing) Co., Ltd., Beijing 102629, China; liuqiang@gobondtest.com

**Keywords:** SARS-CoV-2, neutralizing antibodies (nAbs), binding antibodies, convalescent patients (CP), BBIBP-CorV vaccination

## Abstract

Assessing the duration of neutralizing antibodies (nAbs) following SARS-CoV-2 infection or vaccination is critical to evaluate the protective immunity and formulate public health strategies. In this study, SARS-CoV-2 Ab ELISA (enzyme-linked immunosorbent assay), chemiluminescent microparticle immunoassay (CMIA), as well as pseudovirus neutralization test (PVNT) were performed in two cohorts, convalescent patients (CP) from coronavirus disease 2019 (COVID-19) and BBIBP-CorV vaccinated population. It was found that nAbs and binding antibodies emerged at 14 days post the 1st dose of vaccination, reached peaks at 28 days after 2nd dose vaccination and then gradually declined over time. CP-6M (convalescent patients up to 6 months) from COVID-19 presented stronger nAbs or binding antibodies responses than vaccinees 90 days or 180 days after 2nd dose vaccination. CMIA or SARS-CoV-2 Ab ELISA correlated well with PVNT with high consistency in the two cohorts. It shown that nAbs and binding antibodies can keep 6 months both in CP and vaccinees. Most importantly, our data show the application of using CMIA and SARS-CoV-2 Ab ELISA as rapid screening tests for nAb titer and could be used as alternative strategies for quickly evaluating SARS-CoV-2 nAbs responses in vaccine research.

## 1. Introduction

Globally, as of 14 January 2022, 318,648,834 confirmed cases of COVID-19, including 5,518,343 deaths were reported by WHO. A total of 9,283,076,642 vaccine doses have been administered. Inactivated vaccine BBIBP-CorV (China Sinopharm Bio-Beijing Company) was the first vaccine approved for emergency use by WHO on 7 May 2021and is widely vaccinated in China. All BBIBP-CorV-vaccinated individuals seroconverted successfully by 42 days after the 1st dose vaccination [1]. A phase 3 clinical trial also confirmed the protection of the BBIBP-CorV vaccine with an efficacy of 78.1% reduction of asymptomatic illness [2]. 

Anti-SARS-CoV-2 nAbs could inhibit the binding of virus spike protein to ACE2 on the surface of host cell, thereby blocking viral entry [3,4,5,6]. NAb level has been used to estimate acquired immunity at individual and population level [7,8,9,10,11]. A large multi-center prospective cohort study observed an 84% reduction in the risk of reinfection in antibody-positive population compared with antibody-negative cohort [10]. Thus, evaluating the duration and intensity of nAbs post SARS-CoV-2 infection and vaccination is essential for making public health policies to combat COVID-19. 

Several studies have found that the nAbs titers in CP from COVID-19 could maintain for 6 or even 12 months [12,13,14,15,16,17,18]. However, researches on nAbs titers induced by inactivated vaccines are still limited [1,2,19,20,21,22]. Moreover, the comparison of the nAbs titers between CP and vaccinees may have some guiding significance for the formulation of SARS-CoV-2 vaccine policy.

Many methods for the detection of SARS-CoV-2 nAb have been described previously [17,22,23,24,25], e.g., plaque reduction neutralization test (PRNT), cytopathic effect (CPE) of live SARS-CoV-2 and pseudovirus neutralization tests (PVNT), which are cost- and time-consuming, and cannot be widely implemented [26,27,28,29]. To date, most serology tests mainly detect IgM or IgG antibodies against SARS-CoV-2. Due to the variety of antibody detection methods, the results were always different [24,25,30,31].

Alternative serology assays mainly include the enzyme-linked immunosorbent assays (ELISA) that can detect SARS-CoV-2-specific antibodies and the competition ELISAs that evaluate anti-RBD (receptor binding domain) antibodies competing with host cell receptor angiotensin-converting enzyme 2 (ACE2) [32,33]. After evaluating the sensitivity, specificity and consistency of several methods including RBD-IgG (coated) ELISA, nucleocapsid (N)-IgG (coated) ELISA, WANTAI SARS-CoV-2 Ab ELISA kit and chemiluminescent microparticle immunoassay (CMIA) with PVNT, we selected CMIA and WANTAI SARS-CoV-2 Ab ELISA kits for further study. CMIA can detect antibody responses to RBD against ACE2 receptor by using a competitive-ELISA method. The weaker chemiluminescence value indicates a higher antibody level. It is automated and easy to operate and could tests up to 150 samples in one hour simultaneously. The SARS-CoV-2 Ab ELISA kit uses a double RBD antigen sandwich enzyme-linked immunoassay method to detect antibodies in the sample and the entire experiments process could completed within only two hours. Herein, we explored two simple, rapid and accurate screening strategies, CMIA and SARS-CoV-2 Ab ELISA for predicting SARS-CoV-2 nAbs.

## 2. Results

### 2.1. General Characteristics of Convalescent Patients up to 6 Months (CP-6M) from COVID-19 

**Figure 1:**
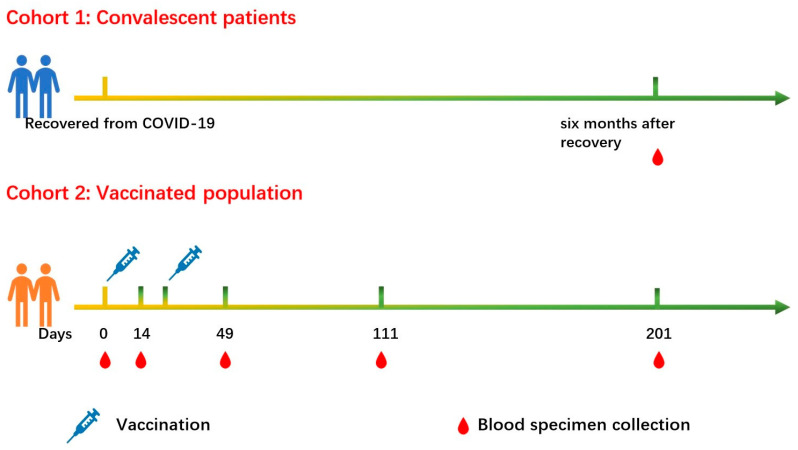
Blood specimen collection and vaccination schedules for the two cohorts. The blood samples were collected from convalescent patients recovered from COVID-19 and vaccinated population. The interval between two vaccinations is 21 days. In the figure, 0 days refers to the day before vaccination; 14 days refers to the days post 1st dose vaccination; 49 days, 111 days and 201 days refers to 28, 90 and 180 days after 2nd vaccination (fully vaccination), respectively. The icons of vaccination and blood sampling were represented by blood drops and syringes, respectively.

**Figure 2:**
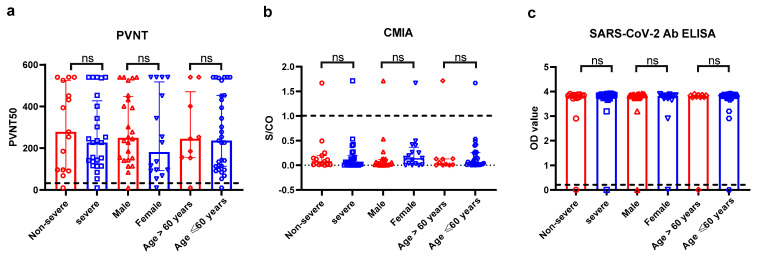
Pseudovirus neutralization test (**a**); chemiluminescent microparticle immunoassay (**b**); WANTAI SARS-CoV-2 Ab ELISA (**c**) were indicated by 50% effective inhibitory concentration, chemiluminescence signal value divided by cutoff (absorbance/cutoff, S/CO) and OD450 value of SARS-CoV-2 RBD-binding antibodies respectively. The dotted line represents the cutoff values of these detections. Two-tailed *p* value were determined using nonparametric Dunn’s Kruskal-Wallis test. The top of boxs display the median and the bars indicate IQR. Each hollow diagram represents a data. ns, no significance.

In cohort 1, 40 individuals who had recovered from COVID-19 approximately six months (158–198 days) were recruited (Figure 1). The clinical data were described in Table 1. Three immunoassays, including CMIA, PVNT and SARS-CoV-2 Ab ELISA were used to detect the levels of binding and neutralizing antibodies in CP. The median (interquartile range, IQR) value of PVNT50 was 240.5 (114–452), S/CO of CMIA was 0.052 (0.01–0.18) and OD450 values of SARS-CoV-2 Ab ELISA was 3.83 (3.77–3.86). The cutoff values of PVNT50, SARS-CoV-2 Ab ELISA and CMIA were 20, 0.19 and 1.00 separately, and nAbs responses showed no difference among subgroups divided by gender, age, and disease severity (Figure 2).

### 2.2. The Seropositivity and Antibody Levels over Times in Vaccinated Population

**Figure 3:**
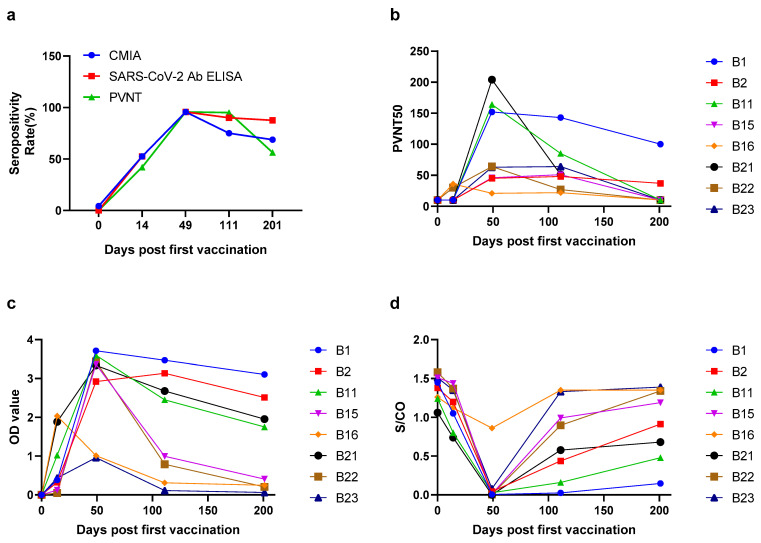
The seropositivity and antibodies level against SARS-CoV-2 in vaccinated population; (**a**) shows the seropositivity of CMIA, SARS-CoV-2 Ab ELISA and PVNT in vaccinated population at a longitudinal follow-up. nAb levels were detected by pseudovirus neutralization test (PVNT) (**b**) and chemiluminescent microparticle immunoassay (CMIA) (**d**); RBD-binding antibody level was detected by SARS-CoV-2 Ab enzyme-linked immunosorbent Assay (SARS-CoV-2 Ab ELISA) (**c**); the various line colors in the picture represent different individuals.

In order to explore the antibody response induced by the BBIBP-CorV vaccination, 23 healthy people (17 females and 6 males, with a median (IQR) age of 27.2 (26.3–39.5) years old) who completed two doses of BBIBP-CorV vaccination were included in cohort 2. PVNT, CMIA and SARS-CoV-2 Ab ELISA were performed before vaccination, 14 days after the first vaccination and 28 days, 90 days and 180 days after the second dose of vaccination, respectively. The time points of vaccination and blood sampling are marked in Figure 1. In this cohort, the seroprevalences of PVNT were as follows: 14 days (42.1%), 28 days (95.7%), 90 days (95.0%) and 180 days (56.3%) after the second vaccination; the seroprevalences of SARS-CoV-2 Ab ELISA were as follows: 14 days (52.6%), 28 days (95.7%), 90 days (90.0%) and 180 days (87.5%); the seroprevalences of CMIA were 14 days (52.6%), 28 days (95.7%), 90 days (75.0%), and 180 days (68.8%) (Figure 3a). Before vaccination, the PVNT50 samples were all less than 20, and the median of OD450 values and S/CO were 0.002 (0.001–0.003) and 1.4 (1.3–1.5), respectively. The results indicated that the population had no neutralization activity and binding ability before vaccination. The antibody appeared at 14 days after the first dose of vaccination, reached peaks at 28 days after the second dose of vaccination and then gradually vanished over time (Table 2, Figure 3). At 180 days post the 2nd immunization, 43.8% and 31.3% of the population did not present nAbs by PVNT and CMIA, respectively. Meanwhile, 12.5% of the population did not show RBD-binding antibodies by SARS-CoV-2 Ab ELISA. Dynamic changes of neutralizing antibody and binding antibody against SARS-CoV-2 were exhibited in Figure 3. 

### 2.3. Comparisons of Antibody Responses in Two Cohorts

**Figure 4:**
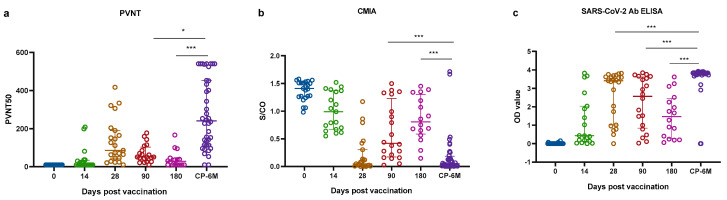
Antibodies response was compared by PVNT, CMIA and SARS-CoV-2 Ab ELISA between vaccinated and convalescent populations. NAb levels were detected by pseudovirus neutralization test (PVNT) (**a**) and chemiluminescent microparticle immunoassay (CMIA) (**b**); RBD-binding antibody level was detected by SARS-CoV-2 Ab enzyme-linked immunosorbent Assay (SARS-CoV-2 Ab ELISA) (**c**); Two-tailed, nonparametric Dunn’s Kruskal–Wallis test was used for multiple comparisons. The bars displayed the median and IQR. Part of the results between groups is shown in the figure. * *p* < 0.05; *** *p* < 0.0001. Each hollow circle represents data.

Next, we then compared the nAbs and RBD-binding antibodies responses between the two cohorts (Figure 4). The nAbs and binding antibodies of the pre-vaccination group were significantly lower than those at 28 days after the second vaccination and the CP-6M group; the *p*-values of three serum immunological assays were all less than 0.0001. In Table 2, the median nAbs titers of the PVNT in the CP-6M group were 2.8-fold that of 28 days after the second vaccination, 4.7-fold that of 90 days after the second vaccination, and 7.8-fold that of 180 days after the second vaccination. The median S/CO value of the CMIA in the CP-6M group was 8.1-fold lower than that of 90 days after the second vaccination, 13.8-fold lower than that of 180 days after the second vaccination. The lower values of CMIA mean higher neutralization activity, the nAbs titers of the CP-6M group were still highest than other groups. The median anti-RBD antibody of the SARS-CoV-2 in the CP-6M group was 1.1-fold that of 28 days after the second vaccination, 1.5-fold that of 90 days after the second vaccination, and 2.6-fold that of 180 days after the second vaccination. The statistical differences were shown among the CP-6M and 90 days or 180 days after the 2nd dose of vaccination by PVNT, CMIA and SARS-CoV-2 Ab ELISA assays (Figure 4). 

### 2.4. The Correlation of Antibody Response between CMIA or SARS-CoV-2 Ab ELISA with PVNT Method

**Figure 5:**
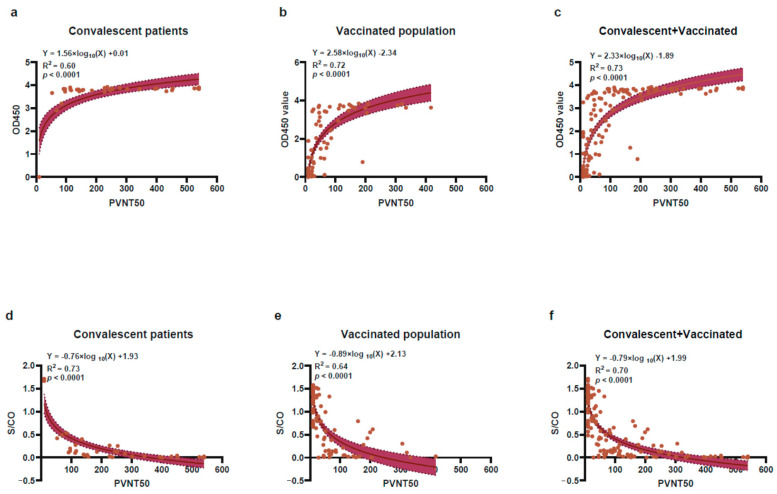
The correlations between PVNT50 with OD450 and S/CO; The correlations between PVNT50 with OD450 values of SARS-CoV-2 Ab ELISA and S/CO of CMIA in convalescent patients (**a**,**d**), vaccinated population (**b**,**e**) and combination of the two cohorts (**c**,**f**) were analyzed by non-linear regression models. A scatter point represents a record, and the brown error band represents the 95% confidence interval. R^2^ represents the degree of linear deviation obtained by fitting the experimental data. The *p*-value tests whether the regression equation is significant. The larger R^2^, the smaller the *p*-value. When R^2^ is greater than 0.5 and *p*-value less than 0.05, this linear model can be considered valuable.

The results of SARS-CoV-2 Ab ELISA and CMIA were further compared between PVNT50 ≥ 20 and PVNT50 < 20 groups (Table 3, Figure 5). Among the convalescent population, the sensitivity, specificity, negative predictive value (NPV), positive predictive value (PPV) and consistency of CMIA and SARS-CoV-2 Ab ELISA are all 100%, which finely correlated with the PVNT results possibly due to a 95% seropositivity rate in this group. The correlation between PVNT50 and S/CO is: S/CO = −0.76 × Log_10_(PVNT50) +1.93, R^2^ is 0.73 and the *p*-value is less than 0.0001; the correlation between PVNT50 and OD450 is as follows: 0D450 = 1.56 × Log_10_(PVNT50) + 0.01, R^2^ is 0.60 and the *p*-value is less than 0.0001(Figure 5a,d). Among the vaccinated population, for SARS-CoV-2 Ab ELISA and CMIA, the sensitivities are 91.8% and 85.7%, the specificities are 80.6% and 83.3%, and the consistencies are 0.73 and 0.69 with PVNT, respectively. The correlation between PVNT50 and S/CO is S/CO = −0.89 × Log_10_(PVNT50) + 2.13, R^2^ is 0.64 and the *p*-value is less than 0.0001; the correlation between PVNT50 and OD450 is OD450 = 2.58 × Log_10_(PVNT50) −2.34, R^2^ is 0.72 and the *p*-value was less than 0.0001 (Figure 5b,e). Combined with the two cohorts, the sensitivities of SARS-CoV-2 Ab ELISA and CMIA are 95.1% and 91.3%, the specificities are 71.1% and 80.0% and the consistencies are 0.70 and 0.71, respectively. The correlation between PVNT50 and S/CO is: S/CO= −0.79 × Log_10_(PVNT50) + 1.99, R^2^ is 0.70 and the *p*-value is less than 0.0001. The correlation between PVNT50 and OD450 values of RBD-IgG titers is OD450 = 2.33 × Log_10_(PVNT50) −1.89, R^2^ is 0.73 and the *p*-value is less than 0.0001(Figure 5c,f). Notably, both SARS-CoV-2 Ab ELISA and CMIA have significant correlations with PVNT no matter in the population of convalescents, vaccinated or two cohorts combined (R^2^ > 0.5 and *p* < 0.05).

## 3. Discussion

The presence of nAbs had been reported correlating closely with protection from infection, reinfection and breakthrough infection with SARS-CoV-2 by several studies [7,10,11]. Thus, understanding the duration and levels of nAbs during SARS-CoV-2 infection and vaccination is essential for scientifically combating COVID-19. In this study, we found that there were no differences in age, gender, and disease stages in antibody responses. This result is consistent with the previous longitudinal study [12,34,35]. Seropositivity rate (99.5%) was found at 6 months after infection [12], and 95.8% was identified in the follow-up 10 months after infection [13]. Similarly, a high nAbs seroprevalence rate (95.0%) was observed in our study. The antibody levels of most convalescent COVID-19 subjects reached a sustained plateau at 6 months and remained stable [12,13,14,15].

Although two doses BBIBP-CorV vaccination provided 100% of seroconversion rate and 78.1% efficacy against symptomatic illness separately, the follow-up duration of the two trials were short, and the median time were only 42 days and 77 days respectively [2,19]. In our study, 200 days follow-up was conducted to demonstrate nAbs dynamics and binding antibodies responses in consecutive samples after SARS-CoV-2 vaccination. Same as convalescent patients, a high seroconversion (95.7%) was showed in vaccinated individuals at 28 days after the 2nd immunization, but PVNT50 in nearly half of this population were below 20 at 180 days after 2nd vaccination. Nevertheless, weak antibody titers do not mean that the vaccine is ineffective. Studies have shown that the nAbs titers of breakthrough infection cases were significantly lower than those in the control group. Infection after vaccination is generally mild or asymptomatic, which may be related to the memory immunity in the body [7,11,36,37].

We also found that the results of CMIA or SARS-CoV-2 Ab ELISA correlated well with PVNT and had a high consistency with the PVNT, no matter in the population of convalescents, vaccinated or two cohorts combined. The excellent results may be due to the antibody detection experiments based on the S antigen or RBD molecules on the surface of SARS-CoV-2. The neutralization mechanism of plasma or antibodies is to block SARS-CoV-2 from entering the host cell by binding to the RBD [23,24,38]. Compared with the 48-hour process of PVNT, these two methods are simple to operate, time-saving, labor-saving and effective, which can be served as alternative methods for quickly evaluating nAbs activity in SARS-Cov-2 infected or vaccinated population. These results were applicable not only to the BBIBP-CorV vaccine, but also to other type vaccines such as mRNA or adenovirus-based vaccines.

Numerous variants of SARS-CoV-2-harboring mutations in spike (both RBD and non-RBD mutations) have been reported in last year, especially the variants of concern (VOCs) e.g., Alpha, Beta, Gamma, Delta and Omicron. Compared with wildtype, a reduction of neutralization activity against VOCs was observed in vaccinees, not only in the BBIBP-CorV, but also in the BNT162b2 or mRNA-1273 vaccines [39,40,41]. For cases infected with the VOCs, commercial kits and pseudovirus neutralization test should be modified on the spike protein/RBD of variants. Therefore, the correlation between SARS-CoV-2 Ab ELISA, CMIA and PVNT mentioned in this paper may need to be further explored for VOCs.

Our study has several limitations. First, although we have conducted at least six months follow-up on populations who have recovered from COVID-19 and vaccinated, the numbers of cases were relatively small. Second, the article was focused to compare nAbs and anti-S-binding antibodies in the recovered and vaccinated populations. The protective mechanisms of nAb has not been studied; however, several studies had already proved the nAbs can prevent future infections in most individuals [7,10]. Third, the research on B lymphocyte immunity has not been carried out, but it is already on the schedule.

## 4. Materials and Methods

### 4.1. Study Participants

Two cohorts were included in this study. Cohort 1 was CP after SARS-CoV-2 infection, and blood samples of 40 patients were collected approximately 6 months (158–198 days). Seven specimens were collected approximately 12 months (355–387 days) after being discharged from the First Affiliated Hospital, Zhejiang University School of Medicine, China. All patients had negative nucleic acid results by reversing real-time polymerase chain reaction (RT-PCR). According to the New Coronavirus Pneumonia Diagnosis and Treatment Program (the 8th edition) published by the National Health Commission of China, clinical classification of COVID-19 pneumonia [42]. We combined mild and moderate patients into a non-severe group and severe and critical patients into the severe group. Cohort 2 included 23 healthy people who have completed two doses of BBIBP-CorV vaccination. All participants had negative nucleic acid results for SARS-CoV-2 by RT-PCR. Informed consent was obtained before all subjects participated in the study. The study was conducted in accordance with the Declaration of Helsinki, and the protocol was approved by the Ethics Committee of First Affiliated Hospital, Zhejiang University School of Medicine (Reference Number 2020433 and 2021376, respectively).

### 4.2. WANTAI SARS-CoV-2 Ab Enzyme-Linked Immunosorbent Assay (ELISA)

The anti-RBD antibodies of SARS-CoV-2 were measured using SARS-CoV-2 Ab ELISA kit and the experimental operation was carried out in accordance with the instructions (Wantai Biological Pharmacy, China). Briefly, 96-well plates were pre-coated with 2019-nCoV-RBD antigen. 100 μL plasma samples were added and incubated 0.5 h at 37 °C and washed. Then, 100 μL HRP labeled 2019-nCoV-Ag were incubated for 0.5 h at 37 °C and washed. Then 100 μL of substrate was added in each well for 15 min at 37 °C. The reaction was stopped by adding 50 μL of 1 nm H_2_SO_4_ to each well, and the reading was taken at 450 nm.

### 4.3. Chemiluminescent Microparticle Immunoassay (CMIA)

The COVID-19 nAbs detection kits (Hotgen, Beijing, China, batch number: 21010115) were based on chemiluminescent microparticle immunoassay (CMIA), which used a competitive-ELISA method to detect nAbs of SARS-CoV-2 in samples. In short, the test was performed by reacting the sample with alkaline phosphatase (ALP)-labeled S-RBD antigen complex. Biotin-labeled receptor protein ACE2 and magnetic microspheres encapsulated with streptavidin were added to promote attachment of the ACE2-S-RBD antigen complex to the magnetic microspheres by specific binding of biotin and streptavidin. Matched automatic chemiluminescence immunoassay analyzer was used to analyze nAb levels, which were presented as the chemiluminescence signal values divided by the cutoff (absorbance/cutoff, S/CO). All operations were carried out in strict accordance with the instructions of the reagent manufacturer. An amount of 100 μL of plasma sample was added, and the whole process took about 30 minutes. The S/CO < 1 was considered positive, S/CO ≥ 1 was negative.

### 4.4. Pseudovirus Neutralization Test (PVNT)

Briefly, 150 µL serial dilutions of human sera (4 serial 3-fold dilutions in Dulbecco’s minimum essential medium (DMEM) with an initial dilution (1:20)) were added into 96-well plates. Then, 50 µL pseudoviruses of SARS-CoV-2(1300 TCID_50_/mL) were added to the plates, followed by incubation at 37 °C for 1 h. Afterward, Hu-h7 cells were added to the plates (1.5 × 10^4^ cells/100 µL cells per well), followed by incubation at 37 °C in a humidified atmosphere with 5% CO_2_. Chemiluminescence detection was performed after 48 hours of incubation. The Reed–Muench method was used to calculate the virus neutralization titers. The result was reported as half maximal inhibitory concentration of PVNT (PVNT50).

### 4.5. Statistical Analysis

A descriptive analysis was carried out using frequencies and median (interquartile range, IQR) quartiles. Two-tailed, nonparametric Dunn’s Kruskal–Wallis test was performed on numerical data, while the chi-square test and Fisher’s exact tests were carried out for the categorical variables. Non-linear regression models were applied to evaluate the correlation between the nAbs and binding antibodies. Graphs and statistical analyses were conducted using GraphPad Prism (version 8.0.2) and SPSS software (version 23.0). *p*-values of less than 0.05 were considered statistically significant.

## 5. Conclusions

In this study, the immune persistency, dynamics of nAbs and binding antibodies were analyzed in convalescents recovered from COVID-19 and vaccinated population. We found that CMIA and SARS-CoV-2 Ab ELISA kits could be used as alternative methods for quickly evaluating nAbs levels in CPs and vaccinees, which is of great significance for assessing vaccine immunization efficacy and herd immunity.

## Figures and Tables

**Table 1:** Clinical characteristics of CP-6M from COVID-19.

Characteristics	All Patients (*n* = 40)
Age, y	
Median (IQR)	52.0 (38.3–59.8)
≤60	30
>60	10
Gender	
Male	24
Female	16
Disease severity	
Non-severe	
Mild	3
Moderate	12
Severe	
Severe	17
Critical	8
Comorbidities	
Liver diseases	19
Hypertension	11
Diabetes	5
Cardiovascular diseases	5
Pulmonary diseases	5
Rheumatic immune diseases	1
Without Comorbidities	9

**Table 2:** Antibody activity in vaccinated population and convalescent patients.

	PVNT, Median (IQR)	CMIA, Median (IQR)	SARS-CoV-2 Ab ELISA, Median (IQR)
Before vaccination	<20 (/)	1.41 (1.26–1.51)	0.002 (0.001–0.003)
14 days after 1st vaccination	<20 (<20–36)	0.99 (0.67–1.37)	0.43 (0.124–2.04)
28 days after 2nd vaccination	86 (45–190)	0.05 (0.01–0.30)	3.41(1.01–3.63)
90 days after 2nd vaccination	51(31–103)	0.42 (0.173–1.229)	2.56 (0.84–3.62)
180 days after 2nd vaccination	31 (<20–42.5)	0.72 (0.59–1.10)	1.47 (0.37–2.31)
Convalescent, 6 months recovered from COVID-19	240.5 (114–452)	0.052 (0.01–0.18)	3.83 (3.77–3.86)

**Table 3:** The detection values of the SARS-CoV-2 Ab ELISA and CMIA in the PVNT50 ≥20 and PVNT50 < 20 groups.

	SARS-CoV-2 Ab ELISA			CMIA		
	Convalescents	Vaccinated Population	Convalescents and Vaccinated Population	Convalescents	Vaccinated Population	Convalescents and Vaccinated Population
Sensitivity	100%	91.8%	95.1%	100%	85.7%	91.3%
Specificity	100%	80.6%	71.1%	100%	83.3%	80.0%
NPV	100%	87.9%	86.5%	100%	81.1%	80.0%
PPV	100%	86.5%	88.3%	100%	87.5%	91.3%
Consistency	1.00	0.73	0.70	1.00	0.69	0.71

NPV: negative predictive value; PPV: positive predictive value; Chi-square test and Fisher’s exact tests were carried out to evaluate the detection values of SARS-CoV-2 Ab ELISA and CMIA in the PVNT50 ≥ 20 and PVNT50 < 20 groups.

## Data Availability

Not applicable.
